# Determination of sub-ps lattice dynamics in FeRh thin films

**DOI:** 10.1038/s41598-022-12602-w

**Published:** 2022-05-20

**Authors:** Michael Grimes, Hiroki Ueda, Dmitry Ozerov, Federico Pressacco, Sergii Parchenko, Andreas Apseros, Markus Scholz, Yuya Kubota, Tadashi Togashi, Yoshikazu Tanaka, Laura Heyderman, Thomas Thomson, Valerio Scagnoli

**Affiliations:** 1 NEST, Department of Computer Science, The University of Manchester, Oxford Road, Manchester, M13 9PL UK; 2 Paul Scherrer Institute, 5232 Villigen PSI, Switzerland; 3 Laboratory for Mesoscopic Systems, Department of Materials, ETH Zurich, 8093 Zurich, Switzerland; 4 Deutsches Elektronensynchrotron, DESY, Notkestrasse 85, 22607 Hamburg, Germany; 5 The Hamburg Centre for Ultrafast Imaging, Universität Hamburg, Mittelweg 177, 20148 Hamburg, Germany; 6 RIKEN, SPring-8 Centre, 1-1-1 Kouto, Sayo, Hyogo 679-5148 Japan; 7 Japan Synchrotron Radiation Research Institute (JASRI), 1-1-1 Kouto, Sayo, Hyogo 679-5198 Japan

**Keywords:** Magnetic properties and materials, Phase transitions and critical phenomena, Surfaces, interfaces and thin films

## Abstract

Understanding the ultrashort time scale structural dynamics of the FeRh metamagnetic phase transition is a key element in developing a complete explanation of the mechanism driving the evolution from an antiferromagnetic to ferromagnetic state. Using an X-ray free electron laser we determine, with sub-ps time resolution, the time evolution of the (–101) lattice diffraction peak following excitation using a 35 fs laser pulse. The dynamics at higher laser fluence indicates the existence of a transient lattice state distinct from the high temperature ferromagnetic phase. By extracting the lattice temperature and comparing it with values obtained in a quasi-static diffraction measurement, we estimate the electron–phonon coupling in FeRh thin films as a function of laser excitation fluence. A model is presented which demonstrates that the transient state is paramagnetic and can be reached by a subset of the phonon bands. A complete description of the FeRh structural dynamics requires consideration of coupling strength variation across the phonon frequencies.

## Introduction

A key challenge in developing advanced materials is to create multi-functional thin films that respond to more than one stimulus. These materials provide a foundation for new types of electronic and computing devices. One example is heat-assisted magnetic recording (HAMR)^1^, where the material responds to both thermal excitation and an applied magnetic field. Practical applications of this effect in data storage require reliable changes in magnetic ordering on a sub-nanosecond timescale. Of particular interest is how quickly and reliably the change in magnetic order occurs. In responding to more than one stimulus the material may undergo changes in the electronic, lattice, and spin structure, and it is necessary to establish how the electrons, phonons, and magnons interact during the transition. In the field of femto-magnetism, such couplings are explored in order to explain the underlying physics^2^.

**Figure 1:**
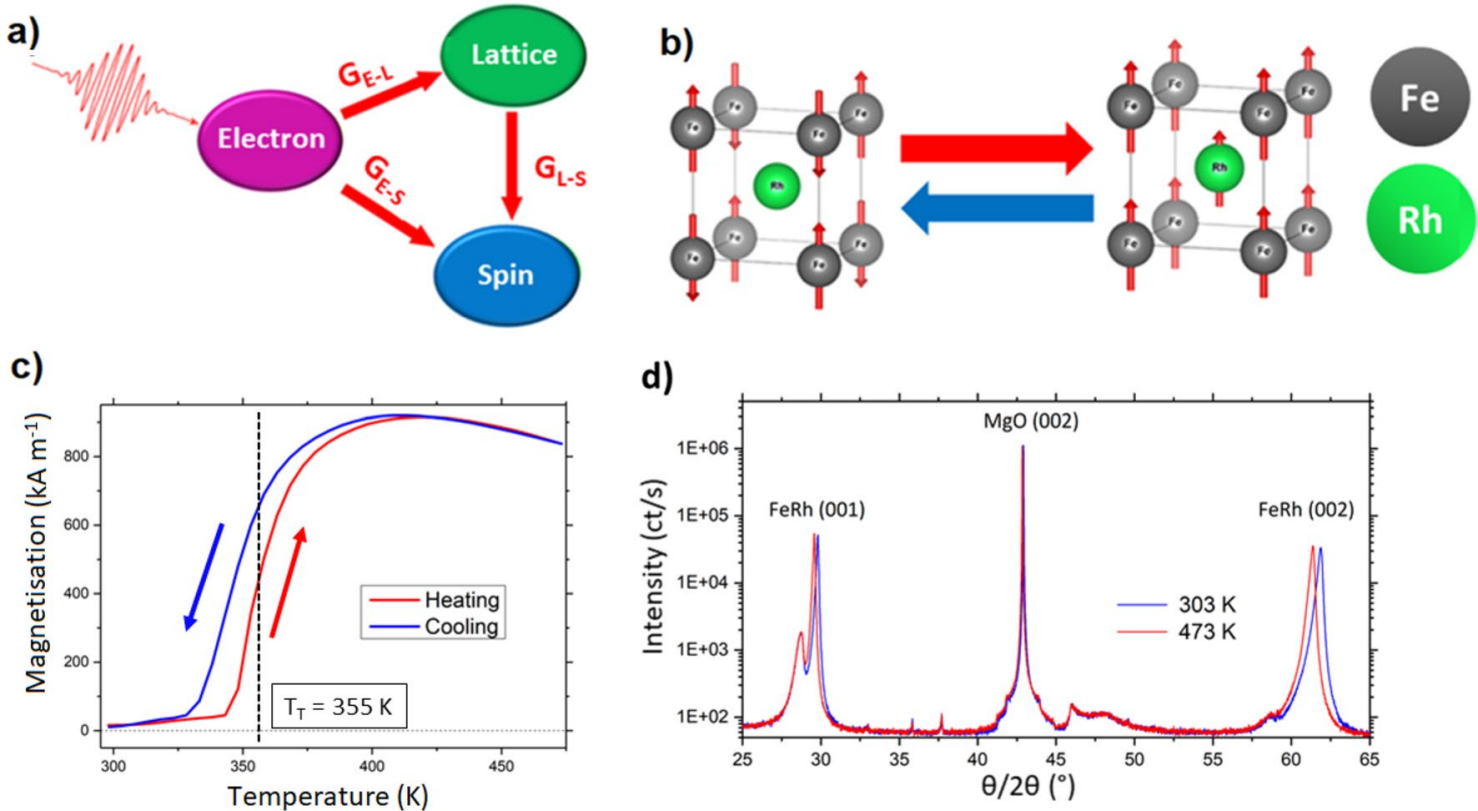
FeRh Phase Transition—(**a**) Three-temperature model first described by Beaurepaire et al.^22^. The excitation is assumed to couple almost immediately to the electron system before relaxing to an equilibrium state via the coupling (G) to the spin and lattice systems. (**b**) Crystal structure and spin configuration of the FeRh B2 ordered phase showing the change in the spin orientation upon heating through the phase transition. Further, in the FM phase strong Fe-Rh hybridisation induces a moment on the Rh site^20^. Characterisation of the FeRh sample used in this experiment: (**c**) The vibrating sample magnetometry (VSM) data shows the evolution of magnetisation, with the transition point at 355 K. The thermal hysteresis is seen from the opening between the heating and cooling cycles. d) The XRD data shows the B2 ordering with the (001) and (002) peaks present. The expansion is seen from the shift in the FeRh peaks upon sample heating.

FeRh is an archetypal system for the investigation of ultrafast behaviour in coupled transitions due to its meta-magnetic phase transition from an antiferromagnet (AF) to a ferromagnet (FM) which occurs at around 380 K^3,4^. Ab initio calculations have shown the ground states of the AF and FM magnetic ordering to be close in energy^5,6^, which means that small perturbations of the system can change the transition temperature^7^. These modelling results have been extensively demonstrated in experiments where it has been found that the transition behaviour can be altered by doping^8^, interfacial strain mediation^9,10^, applied voltage^11^, applied stress^12^, applied field^13^, nanoscale patterning^14^, or ion implantation^15,16^, among other methods. As part of the coupled phase transition shown in Fig. 1b, the electronic structure transforms lowering the resistivity by ≈ 33%^17^, the lattice expands isotropically with a volumetric expansion of ≈ 1%^18^, and the magnetic order changes from a G-type antiferromagnet to a ferromagnet with low coercivity (≈ 300 Oe)^19^. The change in magnetic order of the Fe spins induces a moment on the Rh atom^20^. Due to the coupled nature of the transition, this material has been used to investigate the interaction of the spin, lattice, and electronic systems when it is heated to a non-equilibrium state via laser excitation^21^. The model commonly used to describe such processes is the three-temperature model first described by Beaurepaire et al.^22^ (see Fig. 1a), which connects the electronic, spin, and lattice energies. This is an extension of the two-temperature model^23,24^, where the relaxation to the equilibrium state occurs via the coupling between these three systems, with the assumption that the laser energy is almost immediately transferred to the electrons in the material^25^.

The ultrafast behaviour of FeRh has been investigated with a variety of time-resolved (TR) techniques. The most well-developed of these is TR magneto-optical Kerr effect (MOKE)^5^, showing that the Kerr signal from the FM phase emerges within 100 ps^26^ following excitation with a fs laser pulse. Further investigations using TR photoemission electron microscopy (PEEM) show that the FM domains are established only after 200–500 ps^27^. This delay is associated with the asymmetric transition behaviour of the material^28^ so that AF → FM is not equivalent to FM → AF under time reversal symmetry. This is due to the fact that the AF domains and FM domains are of considerably different size and do not show spatial correlation, hence the creation of AF and FM domains are expected to follow different mechanisms. X-ray magnetic linear and circular dichroism measurements performed across a range of temperatures have shown the asymmetry between the AF → FM and FM → AF pathways^28,29^. When the system heats up, the AF → FM transition occurs by nucleation of FM domains followed by domain growth. In contrast the FM → AF transition occurs solely by a domain nucleation mechanism. The FM domains have a size on the order of 0.1–1 μm and require a time interval of approximately 500 ps before becoming fully established. In contrast, the lattice and electron dynamics occur over much shorter timescales. Valence-band photoemission experiments have shown that the change in the photoemission spectra, following excitation with a fs laser pulse, is fully complete within 5 ps^21^, with transient states observed after 0.4 ps^30^. Furthermore, TR X-ray diffraction (XRD) studies have indicated that the lattice expands with first order dynamics within 10–30 ps^31,32^. A mixed AF-FM phase could be observed within 3 ps, where two Bragg peaks corresponding to the two lattice constants were present. This intermediate speed of transition for the lattice structure indicates that the electron–phonon coupling is stronger than coupling to the spins. According to these measurements, the lattice has already expanded while the spins are undergoing re-orientation.

By measuring TR-XRD, it is possible to monitor the changes that occur to the FeRh lattice after laser excitation and ascertain the presence of transient states on time scales dictated by the excitation laser pulse duration. Extending these measurements beyond the reports of Mariager et al.^32^ is particularly appealing since recent simulations^6,33,34^ of FeRh phonon bands using density functional theory (DFT) have revealed that the lattice dynamics plays a decisive role in the meta-magnetic phase transition, reflected in a significant difference in the expected temperature dependence of lattice vibrations of the ferromagnetic and antiferromagnetic phases.

**Figure 2:**
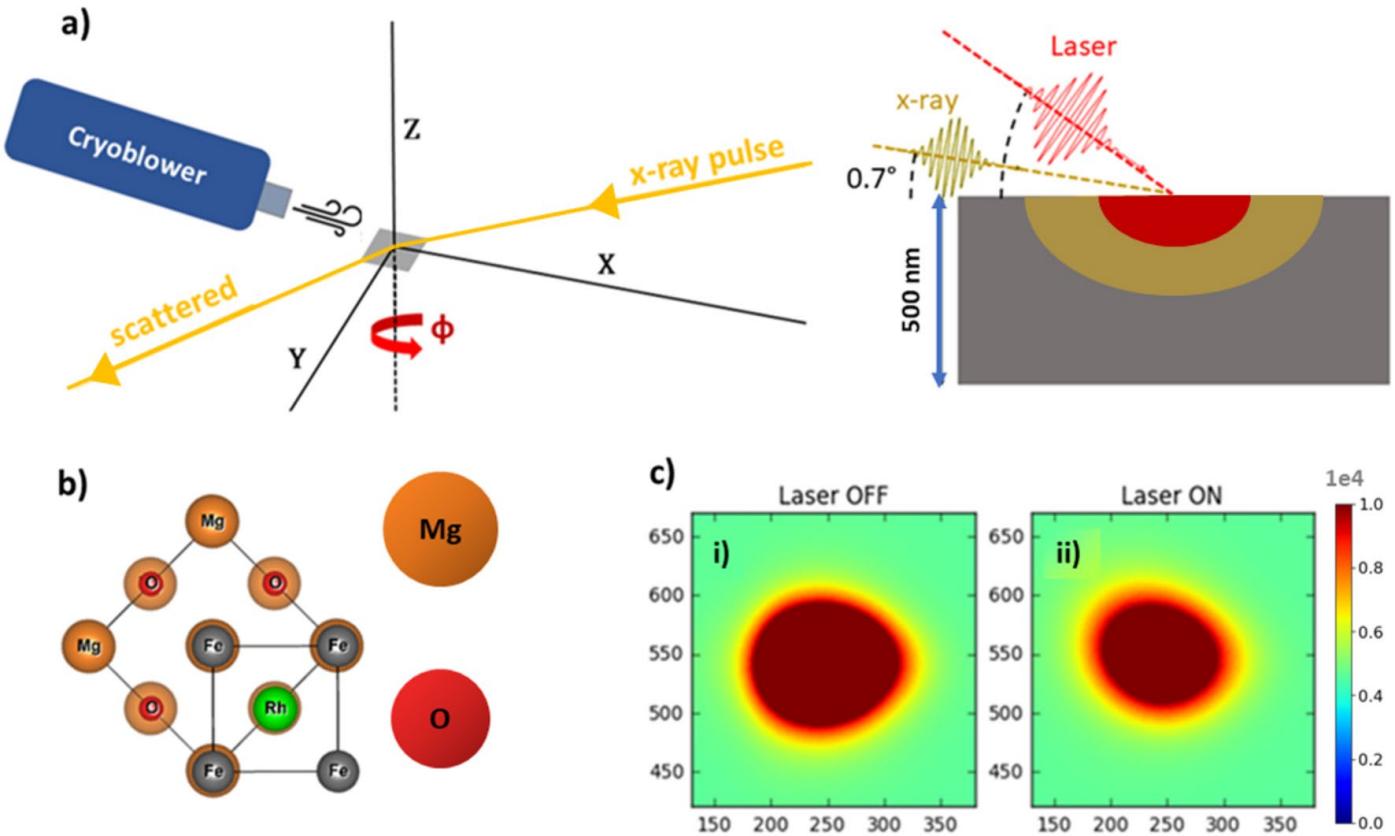
Experimental Set-up—(**a**) Schematic of grazing incidence pump–probe experiment (left panel) with the right panel showing the incidence angles of the pulses where the penetration depth of the laser (red) is 30 nm and the (–101) peak is probed by the X-FEL source (gold). Based on the FeRh refractive index, the X-rays are expected to probe to a depth of 100 nm from the surface of the film at a 0.7° incidence angle, assuming an X-ray attenuation of < 10% by the Pt capping layer (< 3 nm). The strained portion of the film adjacent to the substrate does not contribute to the measured X-FEL diffraction. (**b**) FeRh epitaxial growth on MgO when orientated 45° to [001]. (**c**) Pixel maps of the 2D detector for the region of interest (ROI) around the (–101) diffraction peak at an energy of 6.4 keV for a delay time between the pump and probe of 8 ps. (i) The peak for the unexcited sample is shown, (ii) which is compared to the same peak when the sample is excited by laser fluences of 9.4 mJ cm^-2^.

In this study, we have determined the effect of laser induced heating as a function of fluence as probed by TR-XRD at an X-ray free electron laser (X-FEL) facility, see Fig. 2. As well as the shift in the Bragg peaks indicating the lattice state associated with the FM phase, the intensity of the Bragg peaks will change as a function of pump–probe delay^35,36^. This has been previously used to determine lattice temperature as a function of time by assuming the X-ray intensity is a direct probe of the Debye–Waller factor (DWF)^37^. The lattice constant as a function of temperature as probed by quasi-static XRD measurements provides a reference for the interpretation of this data. The experimental results are compared with a simulation of the transient behaviour using a four-temperature model that demonstrates non-trivial lattice expansion when the system is heated by fluences > 5 mJ cm^-2^.

## Results and discussion

### Temperature dependent static X-ray diffraction

**Figure 3:**
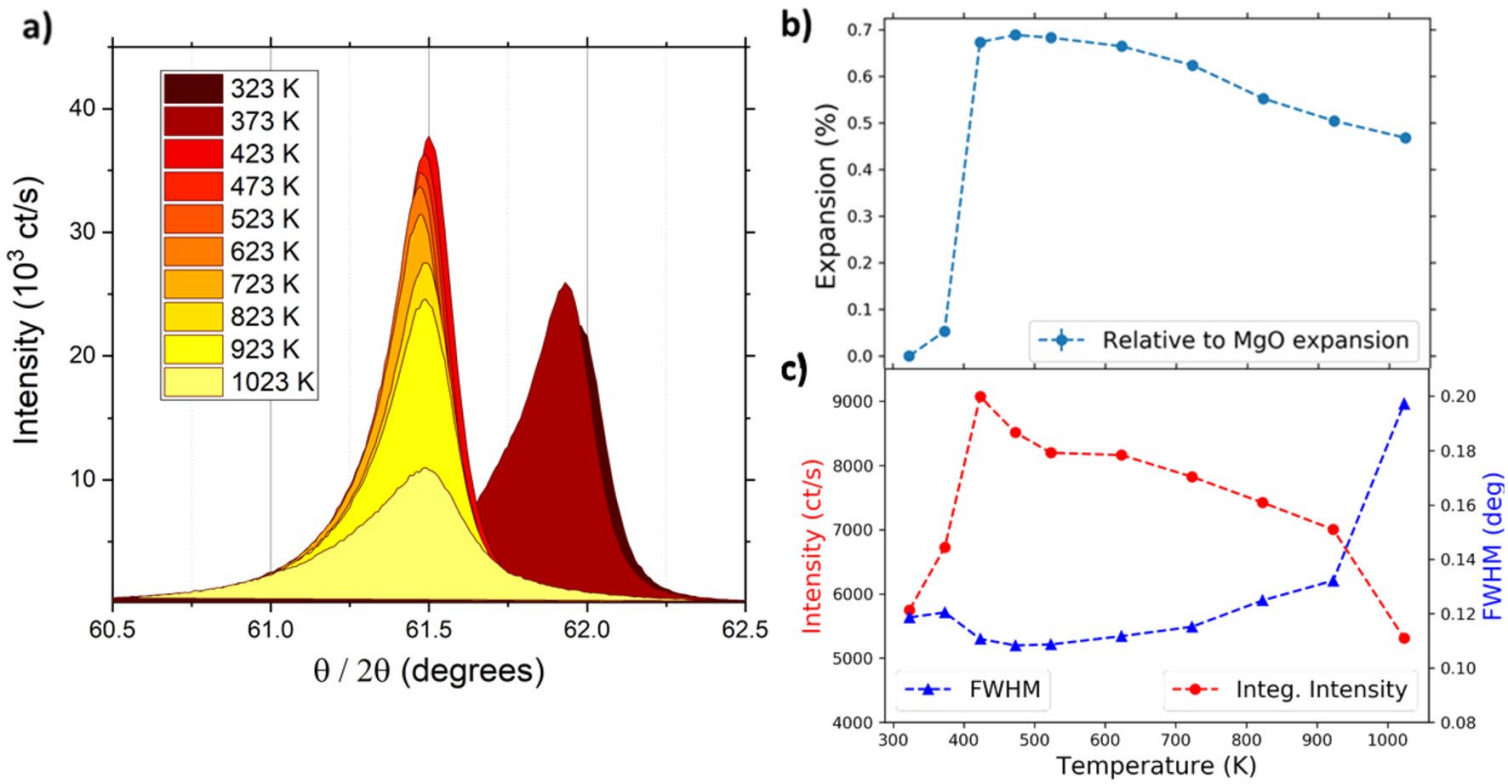
Heated XRD—(**a**) Quasi-static measurement of (002) FeRh peak as a function of temperature determined using an Anton Paar DHS 1100 heated stage on a Rigaku SMARTLAB XRD diffractometer. The sample was maintained under vacuum with a polyether ether ketone (PEEK) dome. (**b**) Lattice expansion of FeRh with respect to the room temperature (002) peak. The MgO (002) peak was used to correct for the substrate induced lattice expansion. The lattice constant was determined by fitting a Voigt function to the data. (**c**) The integrated intensity and FWHM of the (002) peaks extracted from fitting of Voigt function. Error bars from the fit uncertainty are not visible on this scale.

In order to extract quantitative information from TR-XRD experiments, it is essential to have complementary information from quasi-static measurements. It is well known that laser excitation provides a local source of heat. Previous pump–probe experiments on FeRh have shown that laser fluences on the order of 3–5 mJ cm^−2^ instigate the transition to the FM phase. With higher pump fluences, it is possible that the high-temperature paramagnetic (PM) state can be accessed. We therefore determined the lattice properties of FeRh significantly beyond the AF/FM transition with diffraction measurements taken as a function of temperature up to 1023 K, which corresponds to the annealing temperature used following magnetron sputtering deposition. The results gathered on the FeRh (002) diffraction peak are shown in Fig. 3a, for an X-ray energy of 8.048 keV. The lattice expansion as FeRh transitions from AF to FM is seen in the reduction of almost 0.5° in the peak position, from 323 to 423 K. The lattice is then seen to contract slightly as the temperature is increased from 523 up to 1023 K, Fig. 3b. In the data presented, the contributions from the sample alignment and substrate expansion are removed, by taking into account the shift in the MgO (002) peak, which was simultaneously recorded. Further details may be found in the supplementary information (SI). Previous dilatometer work on FeRh powder samples has shown a change in thermal expansion around at 650 K^38^, which was presumed to be a signature of the Curie temperature-T_C_. Consequently, we attribute the lattice contraction above 650 K to be a signature of the PM phase.

Additional information about the structural evolution of the FeRh lattice is available from the intensity and FWHM of the XRD peak (Fig. 3c). The intensity (FWHM) shows a continuous non-linear reduction (increase) at the highest temperatures measured, resulting from increased thermal motion, typically parametrized with the DWF via the atomic displacement **u**_n_. Quantitatively, using the Einstein correlated model^39^, the temperature of the lattice (T_L_) will, result in a reduction of the peak intensity I(**q**,T_L_) (with reciprocal lattice vector **q**) given by^36^

1 $$\begin{array}{*{20}c} {I\left( {{\mathbf{q}},{\text{T}}_{{\text{L}}} } \right) \propto \left( {{\text{e}}^{{ - \left( {{\mathbf{q}} \cdot {\mathbf{u}}_{{\text{n}}} } \right)^{2} }} } \right)} \\ \end{array}$$

assuming an isotropic harmonicity of the scattering centres with average atomic displacement

2 $$\begin{array}{*{20}c} {\left\langle {\text{u}}^{2} \right\rangle} = \frac{{9\hbar^{2} }}{{{\text{M}}\;{\text{k}}_{{\text{B}}} \Theta_{{\text{D}}}^{2} }}{\text{T}}_{{\text{L}}} \\ \end{array}$$

where M is the unit cell mass, and Θ_D_ is the FeRh Debye temperature with ħ and k_B_ the reduced Planck’s and Boltzmann constants, respectively. Therefore, from the data in Fig. 3 we can determine the DWF of our sample and use the inferred values to estimate the lattice temperature^40^ in the time-resolved XRD measurements described in the next section.

### Time-resolved X-ray diffraction

Following the seminal time-resolved X-ray diffraction experiment of Mariager et al.^32^ and recent theoretical predictions^34^ of a significant difference in the expected temperature dependence of lattice vibrations of the FM and AF phases, we performed a X-FEL based time-resolved experiment to determine the FeRh AF and lattice dynamics on the sub-ps time scale, at an X-ray energy of 6.408 keV.

**Figure 4:**
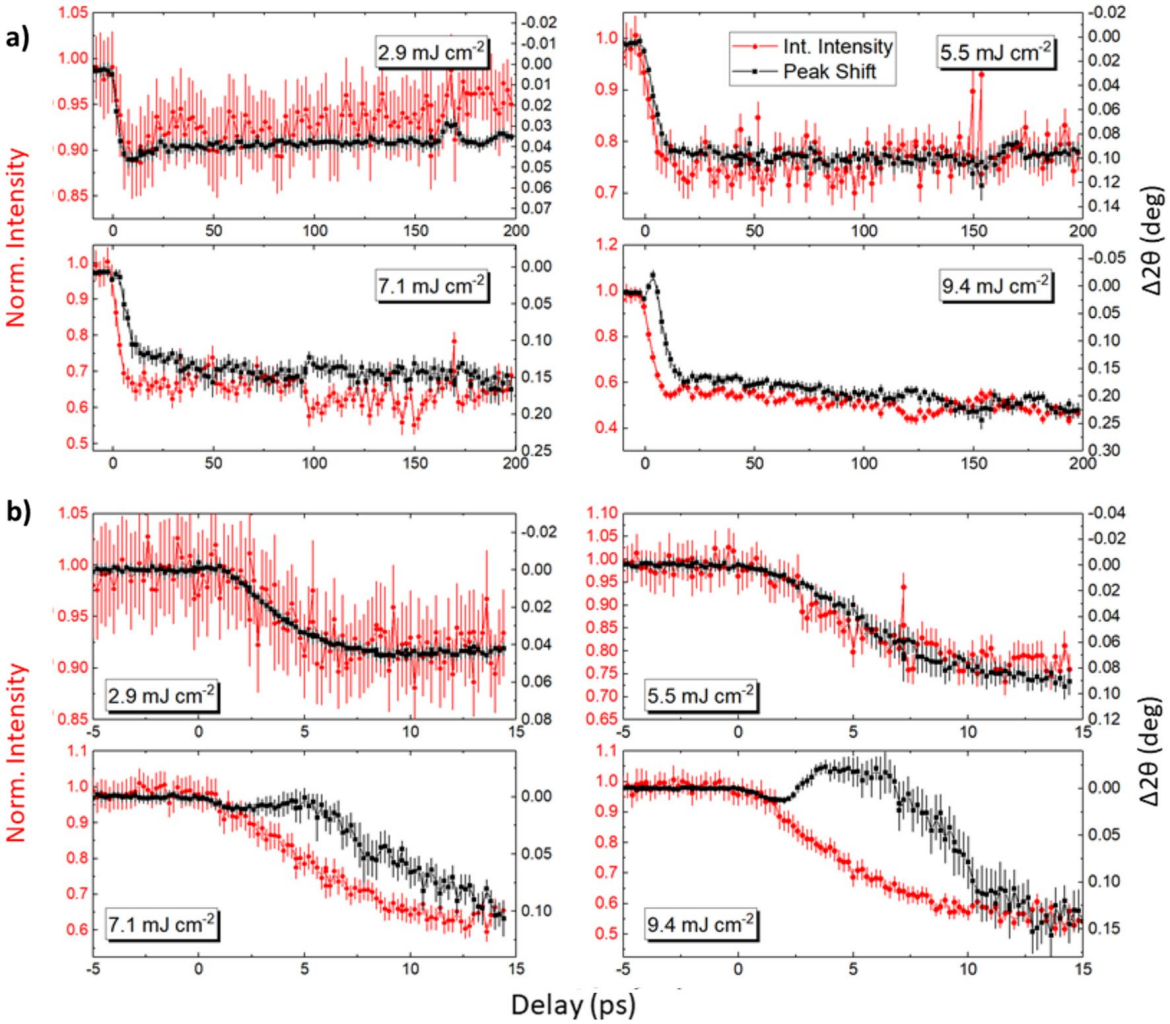
TR-XRD of the (–101) FeRh Peak—(**a**) Peak intensity and 2θ shift as a function of probe delay for the FeRh (–101) XRD peak. The extracted quantities show similar dynamics, initially decaying within 10–30 ps and recovering over 100’s of ps. (**b**) The same quantities focusing on the initial excitation up to 15 ps. For fluences 5.5 mJ cm^-2^ and above, the peak shift shows different dynamics to that of the intensity, with increasing divergence for increasing fluence.

The FeRh film was excited with laser pulses of various fluences. The scattering geometry, shown in Fig. 2a, was optimized to achieve similar laser and X-ray probe depths^41^. This grazing incident geometry ensures that only the region of the film farthest from the substrate is probed which minimises the effect of substrate clamping allowing essentially isotropic expansion of the FeRh. Further details can be found in the SI. Examples of (–101) diffraction peaks as measured by the 2D detector are shown in Fig. 2c, with pixel maps shown for both the excited and unexcited sample, in the presence or absence of laser illumination, respectively. The normalised intensity refers to the change in the integrated peak intensity within the ROI shown in Fig. 2c. The peak position was found by a centre of mass (COM) fit to the intensity of the peak. From this it was possible to monitor the lattice expansion of the sample within the laser pumped region. Figure 4a shows data over the entire range of time delays measured, while Fig. 4b concentrates on the initial 15 ps. The observed shift in the position of the signal COM is due to the lattice expansion, whereas the change in the peak intensity can be used to infer the lattice temperature via the DWF^35,36,39^. At a pump fluence of 2.9 mJ cm^-2^ the intensity and peak shift show similar dynamics, with a maximum change observed within 10 ps followed by a gradual relaxation over 100’s of ps. Such behaviour is similar to that previously reported by Mariager et. al.^32^ In this case, the FM phase is found to emerge with a growth lifetime (τ_G_) of 6 ± 1 ps as calculated by modelling the data with a growth-decay model adapted from Radu et. al.^26^ for t > 0, given in Eq. (3):

3 $$\begin{array}{*{20}c} {\Delta I\left( {\text{t}} \right) = A\left( {1 - {\text{ e}}^{{{\raise0.7ex\hbox{${ - {\text{t}}}$} \!\mathord{\left/ {\vphantom {{ - {\text{t}}} {{\uptau }_{{\text{G}}} }}}\right.\kern-\nulldelimiterspace} \!\lower0.7ex\hbox{${{\uptau }_{{\text{G}}} }$}}}} } \right) + B\left( {{\text{e}}^{{{\raise0.7ex\hbox{${ - {\text{t}}}$} \!\mathord{\left/ {\vphantom {{ - {\text{t}}} {{\uptau }_{{\text{R}}} }}}\right.\kern-\nulldelimiterspace} \!\lower0.7ex\hbox{${{\uptau }_{{\text{R}}} }$}}}} } \right)} \\ \end{array}$$

where ΔI(t) is the change in the scattering intensity, A and B are fitting constants, t is the delay between the pump and probe pulses, and τ_G_ and τ_R_ refers to growth and the relaxation lifetimes of the transient FM phase. However, as the fluence is increased the peak position and the intensity do not follow the same time evolution.

First, we discuss intensity as a function of probe delay. On short time scales (t < 30 ps), the intensity for all fluences, decays with a characteristic time constant of τ_G_ ~ 6–12 ps, reflecting the laser induced temperature increase of the sample. Over the longer timescales seen in Fig. 4a, the FM lattice state relaxes with τ_R_ ~ 80–120 ps. For fluences > 5 mJ cm^-2^, a continuous decrease in intensity is instead observed with a similar time scale (τ ≈ 100 ps). This indicates the existence of a different sample heating regime, which we associate with heat dissipation occurring from the laser excitation centre to the unexcited sample volume probed by the X-rays (given that they interact with a larger sample volume than is excited by the laser pulse).

**Figure 5:**
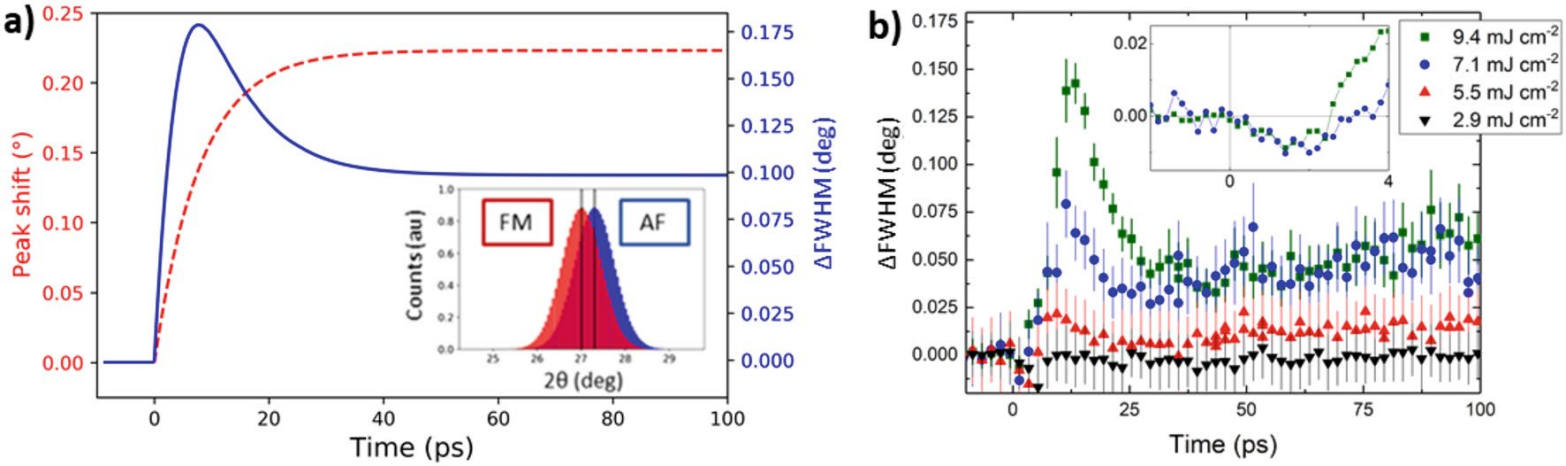
FWHM of TR-XRD Peak—(**a**) Model showing how the FWHM of the FeRh (-101) peak is expected to change with probe delay following laser heating to 400 K. The transition is first order and the ratio of AF:FM peak intensity changes with temperature according to the ratio of the two phases based on the magnetometry data of Fig. 1c. The inset shows the expected intensity of the AF and FM peaks several ps after laser excitation. The peak shift and change in FWHM are extracted from a Gaussian fit to the summation of the peaks. (**b**) Change in FWHM of (–101) peak as a function of probe delay for different laser fluences. The initial peak in the data points to the greatest mixing of the two phases. A longer trend is seen for higher fluences which we ascribe to thermal diffusion in the sample. We note that, irrespective of laser fluence, a small drop in the FWHM is observed in the first few ps after laser excitation, inset for the two highest fluences.

Next, the peak position is discussed, which shows more complex behaviour in the sub 15 ps region. Specifically, for the highest fluences a non-monotonic behaviour can be observed. Due to the width of the measured peaks resulting from the spectral width of the ‘pink’ beam^42^ (ΔE = 40 eV ≈ 0.2°), the position (and intensity) of the two peaks, signalling the coexistence of the AF and FM phase, cannot be directly resolved. However, the peak centre of mass can be used to estimate the ratio of the AF and FM phase, assuming first-order transition dynamics, if the AF/FM volume fractions as a function of temperature are known^32^ (see Fig. 1). In order to construct a model to explain how lattice dynamics affect the peak position and FWHM after the laser pulse excitation, we have estimated the percentage of each phase as a function of temperature from the VSM data presented in Fig. 1c. Assuming the maximum saturation magnetisation corresponds to entirely transitioned FeRh, the relative moment at a given temperature is equated to the percentage of FM phase. The laser was assumed to heat the lattice according to a simple thermal model where the rate was based on the observed lattice dynamics (τ_G_ ≈ 10 ps at high fluences) estimated by Eq. (3). Based on the percentage of each phase, the scattering intensity of both peaks (each with a FWHM of 0.45°) could be estimated and used to describe the observed peak position shift as a function of time (see SI for further details). The intensity of each peak is assumed to be in direct proportion to the phase volume. Additionally, we could observe how the FWHM of this simulation evolves by fitting a Gaussian function to the combined peaks as illustrated in the inset of Fig. 5a. By comparing the form of the FWHM curves from the first-order simulation (Fig. 5a) and the experimental results (Fig. 5b), we see that the model reproduces the asymptotic peak shift occurring on long time scales as well as the major change in the FWHM. Therefore, we conclude the FeRh lattice expansion follows first-order dynamics, as previously shown by Mariager et al.^32^.

However, the model does not capture non-monotonic behaviour of the FWHM that occurs in the first few ps (Fig. 5b inset). In particular, there is a reduction in the FWHM immediately following laser excitation that recovers within 5 ps. For this short-lived period, the sample has a narrower diffraction peak than in the unexcited state. Assuming the transient FWHM behaviour can be attributed purely to the changes in the sample structure, this indicates the system becomes more ordered on a sub-ps timescale following laser excitation, which is counterintuitive. In order to understand this observation, we utilize the static XRD results shown in Fig. 3. The FWHM of the (002) peak decreases when the sample is heated above 350 K, see Fig. 3c. This is consistent with the release of inhomogeneous strain across the film by the mixed AF/FM phase, where substrate induced strain acts to expand the lattice^43^.

Further instances of non-monotonic behaviour are the complex peak shift dynamics shown in Fig. 4b. The data from the initial 5–10 ps indicates that a lattice contraction is competing with the volumetric expansion of the FeRh film. To explain this behaviour, we include another growth-decay term in our model with much faster dynamics and opposite sign. This assumes there are two channels through which the electron-lattice coupling occurs with independent growth lifetimes; the usual channel which acts to expand the lattice and a transient mode which acts in opposition. This transient state is characterised by adding another term to Eq. (3) of magnitude C, with independent τ_G_* and τ_R_* as follows:

4 $${ }\begin{array}{*{20}c} {\Delta 2\theta \left( {\text{t}} \right) = A\left( {1 - {\text{ e}}^{{{\raise0.7ex\hbox{${ - {\text{t}}}$} \!\mathord{\left/ {\vphantom {{ - {\text{t}}} {{\uptau }_{{\text{G}}} }}}\right.\kern-\nulldelimiterspace} \!\lower0.7ex\hbox{${{\uptau }_{{\text{G}}} }$}}}} } \right) + B\left( {{\text{e}}^{{{\raise0.7ex\hbox{${ - {\text{t}}}$} \!\mathord{\left/ {\vphantom {{ - {\text{t}}} {{\uptau }_{{\text{R}}} }}}\right.\kern-\nulldelimiterspace} \!\lower0.7ex\hbox{${{\uptau }_{{\text{R}}} }$}}}} } \right) - C\left( {1 - {\text{ e}}^{{{\raise0.7ex\hbox{${ - {\text{t}}}$} \!\mathord{\left/ {\vphantom {{ - {\text{t}}} {{\uptau }_{{\text{G}}} {*}}}}\right.\kern-\nulldelimiterspace} \!\lower0.7ex\hbox{${{\uptau }_{{\text{G}}} {*}}$}}}} + {\text{ e}}^{{{\raise0.7ex\hbox{${ - {\text{t}}}$} \!\mathord{\left/ {\vphantom {{ - {\text{t}}} {{\uptau }_{{\text{R}}} {*}}}}\right.\kern-\nulldelimiterspace} \!\lower0.7ex\hbox{${{\uptau }_{{\text{R}}} {*}}$}}}} } \right)} \\ \end{array}$$

**Figure 6:**
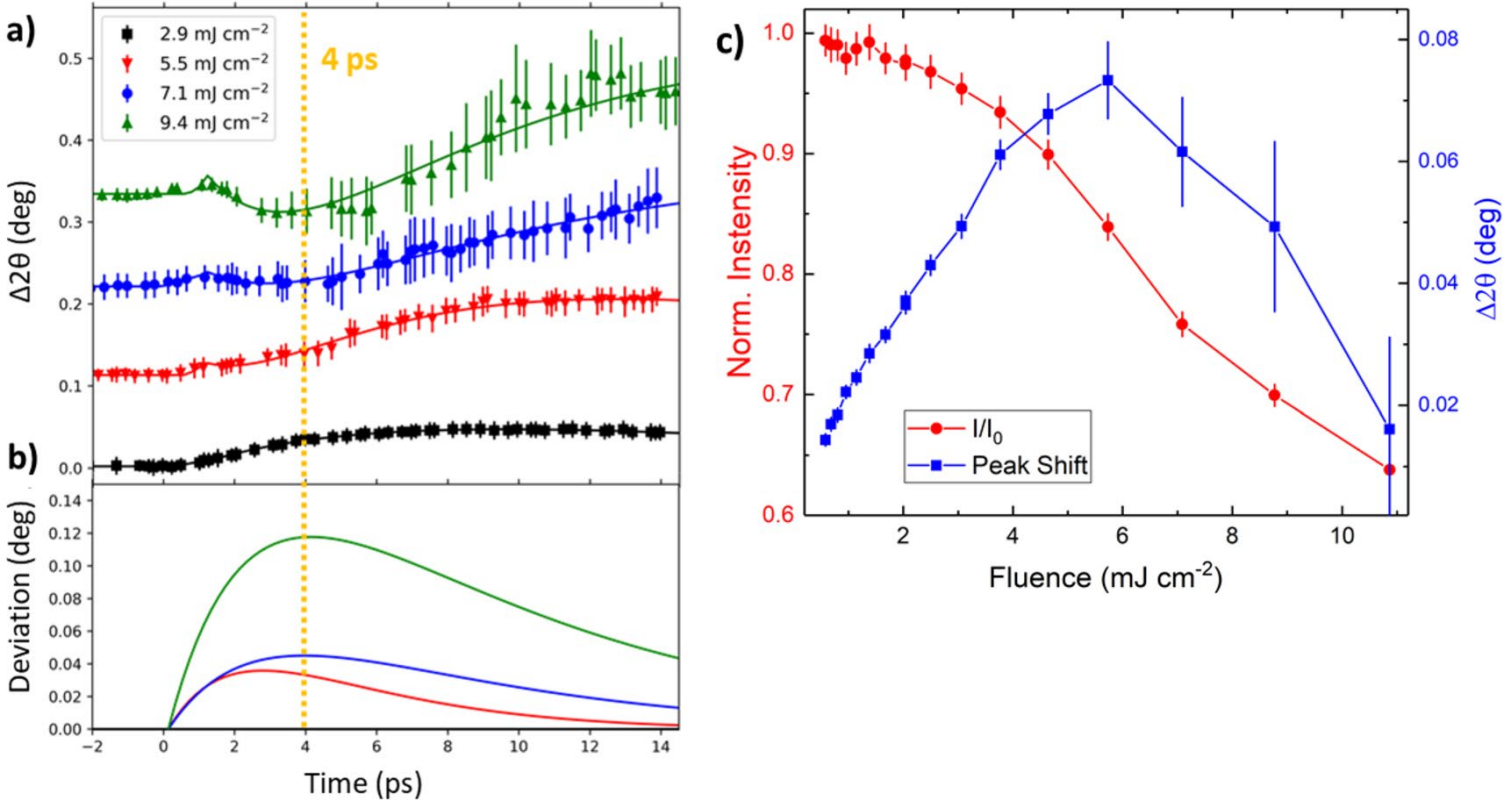
Transient Lattice State—(**a**) The shift of the (–101) FeRh peak as a function of probe delay. Data is offset vertically for clarity. Error bars represent the standard deviation of the intensity of the individual measurements when binned according to a jitter correction procedure. Equation (4) was fitted to the data shown by the full lines. (**b**) The transient term that acts to contract the lattice is plotted using the parameters obtained from fitting the data. This shows that higher laser fluences result in a stronger and longer-lived transient state with τ_G_* ≈ 3 ps and τ_R_* ≈ 6 ps. (**c**) Peak shift and change in intensity of (–101) FeRh Bragg peak as a function of laser fluence, at a fixed time delay of 4 ps. The increasing pump fluence causes a steady decrease in intensity. The reduction in peak shift above 5 mJ cm^-2^ is assumed to be due to the induced transient state.

This state disappears within 10–15 ps, which indicates a short relaxation lifetime and by extension, strong coupling. Using Eq. (4), the peak shift as a function of delay time could be described (see Fig. 6a). Figure 6b shows a plot of the estimated contribution of this transient state to the peak shift and demonstrates how this term strongly depends on the laser fluence. There is an increase both in the intensity (C) and lifetime (τ_R_*) with increasing fluence seen from the larger peak deviation, indicating a transient state that is more dominant with greater laser heating. The short lifetime of this state is not consistent with a strain wave, as propagation through a 500 nm film would result in a mode with period on the order of 200 ps, based on the reported speed of sound in FeRh^32,44^ (ν = 5.1 km s^-1^).

Having established the presence of such a transient lattice state, we measured the change in intensity and peak shift of the (–101) peak as a function of laser fluence at a fixed delay of 4 ps (see Fig. 6c), in order to determine the laser fluence required to excite this state. Contrary to the expectation that there should be an asymptotic peak shift since the full AF → FM transition is induced by increased laser heating, we instead observe a change in behaviour for fluences greater than 5 mJ cm-^2^. Above this fluence value, the peak shift starts to decrease rather than reaching an asymptotic value. The extracted intensity of the observed peaks suggests that the sample is significantly heated, which is likely to be beyond the AF-FM transition temperature, and approaches the high temperature PM state^38,45^.

### Accessing PM phase via a transient phonon state

The non-monotonic behaviour shown in Fig. 6a provided motivation for the construction of a more accurate model to describe the lattice expansion. The three-temperature model^22^ presented in Fig. 1a is suitable only to describe the dynamics of the Bragg peak intensities, providing an estimate of the overall coupling strength (G_E-L_) between the electron and phonon systems^46,47^.

Above a laser fluence of 5 mJ cm^-2^, the dynamics diverge from those predicted using such a collective coupling model_._ This divergence may be captured by including a transient phonon state in the three-temperature model in the form of an intermediate lattice coupling. This is described in the review article of Johnson et al.^35^ as a 4th temperature which provides a competing pathway for relaxation to the equilibrium state^36,48^. Through the four-temperature model, both growth lifetimes (τ_G_ and τ_G_*) of Eq. (4) are included. The transient lattice state with faster growth dynamics (τ_G_^*^) is assumed to be a ‘hot phonon’ channel^47^ that is heated more efficiently than the lattice as a whole. Building on this hypothesis we propose that, by further pumping the sample, it is possible to increase the lifetime of the transient state in Fig. 6b. By adapting the three-temperature model, we can infer the lifetime of such a ‘hot phonon’ state as a function of fluence.

The modified three-temperature model was realised by assuming stronger interaction between the electron system and a particular subset of the phonon bands and modelling how this would affect the evolution of the lattice temperature. This assumption requires optical phonon modes that can couple more strongly to the electrons than the anharmonic acoustic phonons^35,36^—the main contributors to the DWF below Θ_D_^37^ (≈ 360 K for AF FeRh^49^). A recent TR-photoemission study proposed changes in the electronic structure occur via charge transfer from Fe to Rh^30^, implying stronger electron coupling for such phonon modes. Examples of optical phonon branches along the [110] direction of FeRh have been explored in detailed ab initio and spin-polarised calculations using density functional theory (DFT)^33,50^. Here, nuclear resonant inelastic X-ray scattering provides a reference for the density of states (DOS)^33^ along these phonon branches in the AF phase. Modelling the relaxation dynamics of the film according to the transient ‘hot phonon’ model of Mansart et al.^36^ allows a theoretical estimate of the lattice temperature to be obtained. This model incorporated a 15% fraction of efficiently coupled phonon modes, corresponding to the relative DOS of transverse optical phonons^33^. Coupling parameters were estimated as the inverse of the growth lifetimes derived from data shown in Fig. 6a. The thermal properties of FeRh were found from calorimetric data in the literature^49,51,52^. It was assumed that the FM phase occurs between 355 and 700 K based on VSM measurements shown in Fig. 1c, while the PM phase exists above 700 K based on a stoichiometric ratio of Fe_50_Rh_50_^53^ and the quasi-static XRD measurements shown in Fig. 3a. The differential equations describing this model can be found in the SI.

**Figure 7:**
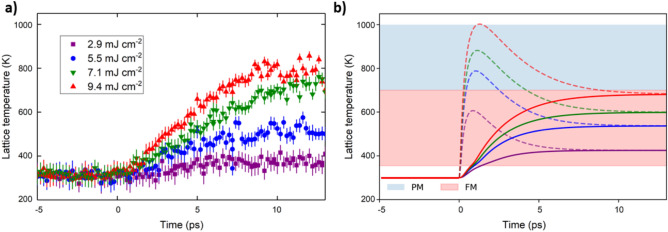
Transient Lattice Temperature—(**a**) Lattice temperature as a function of pump–probe delay based on the intensity of the transient XRD peaks. The extracted Debye temperature, Θ_D_ of FM FeRh was used to estimate the transient temperature. The uncertainty in the lattice temperature is a combination of the standard error in the transient intensity and uncertainty from the DWF fit procedure. (**b**) The evolution of the lattice temperature as derived from the hot phonon model of electron–phonon coupling. Dotted lines refer to the transient phonon states which are assumed to be a subset of the optical phonon modes, estimated to be 15% of the total phonon population. The full lines indicate the average lattice temperature when the entire phonon system is considered. This model does not consider heat dissipation from the excitation centre.

Figure 7a shows the lattice temperature obtained from the intensity of the transient (–101) peaks estimated using the DWF extracted from the quasi-static measurements of the (002) peak (see SI, Fig. S5). Figure 7b presents the simulated transient temperatures of the highly-coupled phonon modes (dotted lines) and the total lattice (full lines). We see that the simulated average lattice temperature in Fig. 7b compares well to the extracted temperature profile shown in Fig. 7a. The lattice is predicted to reach temperatures of 680 K in the case of highest fluences within 10 ps. The addition of an intermediate phonon state with stronger electron-lattice coupling (G_E-L_*) captures the divergence from the expected behaviour over ps timescales. Using this additional phonon channel, we demonstrate how the PM state can be reached within 5 ps in Fig. 7b. This transient PM state has a strong fluence dependence above 5.5 mJ cm^-2^, with greater PM character and longer lifetimes as fluence is increased. This compares well to short-lived lattice dynamics we observe in the peak position (Fig. 6b) assuming the volume contraction is associated with the PM phase. The simulations and fitting of the data indicate the transient PM phase can be excited at high laser fluences with a lifetime, τ_R_ ≈ 6 ps. As such, the volumetric expansion of FeRh is non-trivial with these results demonstrating how higher temperature phases contribute to the transient behaviour. The four-temperature model presented here indicates that the electron-lattice coupling is not constant across all phonon bands. The understanding of the lattice dynamics that we have gained here will contribute to an improved description of the FeRh coupled phase transition^7^.

## Conclusions

The sub-ps capabilities of the SACLA X-FEL^54,55^ has allowed us to investigate the ultrafast behaviour of the FeRh lattice upon laser excitation. We have shown the lattice dynamics of the FeRh volumetric expansion are not trivial across all fluences of laser excitation. Indeed, the system shows non-trivial dynamics at high fluences which heat the sample significantly beyond the AF-FM transition temperature. This was compared to the quasi-static behaviour of the Bragg peaks measured using conventional heated XRD. The intensity and 2θ position of these peaks could be used to describe the lattice temperature and expansion as a function of pump–probe delay with a temporal resolution not previously reported. This resulted in a perturbation to the expected dynamics at high laser fluence where the lattice contracts before finally expanding as initially predicted. While we cannot comment on the exact mechanism of this state, based on previous investigations on structural dynamics we demonstrate that a four-temperature model using a transient lattice state maps the observed behaviour. Such behaviour suggests the system relaxes through a subset of the optical phonon bands which are highly coupled to the electronic system. These results provide important evidence for the inclusion of the PM phase in analysing the laser-induced phase transition of FeRh. The model developed provides for better understanding of the longer dynamics describing electron-spin and lattice-spin coupling in this material.

## Methods

### Sample preparation

The samples investigated were nominally 500 nm thick Fe_50_Rh_50_ films dc magnetron sputter deposited on MgO substrates from an equiatomic FeRh alloy target. The films were deposited using an AJA ATC-2200 sputter system and, following deposition at 650 °C, the samples were annealed for 2 h at 750 °C^56^. After cooling to room temperature, a Pt cap (3 nm) was deposited to inhibit surface oxidation. The annealing step is important to achieve the necessary B2 crystal structure for the required meta-magnetic state^56^. Further details of the deposition can be found in previously published work^57,58^. The sample was prepared for X-ray measurements by cutting it into a 5 × 5 mm square using a diamond saw. A photoresist (PMMA) layer was deposited prior to cutting to prevent film damage and flaking before subsequent removal using acetone and isopropyl alcohol. The sample dimensions were chosen to be compatible with the mounting stage of the goniometer used in the X-FEL facility.

### Sample characterisation

Characterisation data for the sample is presented in Fig. 1. The crystalline structure of the FeRh films was confirmed by X-ray diffraction (XRD) analysis (Fig. 1d). The film is found to align in-plane at 45° to the cubic MgO substrate (see Fig. 2). This fulfils the lattice matching condition with a mismatch of < 2% for planes FeRh[001](011)||MgO[001](001). The volumetric expansion of the FeRh through the meta-magnetic phase transition was measured to be 0.75% when heated from 30 °C to 220 °C, with the expansion in the MgO lattice being 0.01%. Vibrating sample magnetometry (VSM) confirmed that the meta-magnetic transition occurs at 355 K, with maximum saturation magnetization Ms of 920 kA m^−1^ (at a temperature of 413 K) as shown in Fig. 1c.

Structural Analysis: Quasi-static XRD measurements on the FeRh sample were undertaken on a Rigaku SMARTLAB diffractometer equipped with an Anton-Paar DHS 1100 heated stage. A PEEK dome was used to maintain a pressure of 10^–2^ mbar and reduce sample oxidation. Alignment of the samples followed the pre-installed procedure on the diffractometer and the sample was allowed to thermalise after each heating step. Scattering from the dome was reduced by scanning a region of 2θ with no PEEK diffraction peaks, about FeRh (002).

### Pump–probe crystallography

TR-XRD measurements were carried out on BL 3 of SACLA^54,55^ in Japan with the experimental geometry shown in Fig. 2a. The sample temperature was raised using a pulsed laser excitation with wavelength 800 nm, pulse duration of 35 fs, and an elliptical spot size of 480 × 500 μm^2^. The angle of incidence used for the pump laser was 15.4°, also corresponding to the angle between the incoming X-rays and the laser as shown in Fig. 2. The crystal orientation was found by scanning rocking curves of the (001) and (111) peaks of the FeRh. The corresponding orientation matrix was used to calculate the relevant goniometer angles for the (–101) Bragg peak with the required grazing incidence. The scattered x-rays were detected using a 2D detector which was fixed in position while the angle ϕ (about surface normal) was swept to provide the rocking curves. The 2D detector was a multi-port charge coupled device (MPCCD) having 512 × 1024 pixels^59^. Shot-to-shot variance in the X-FEL pulse arrival time is accounted for using an arrival timing monitor^60^. This jitter correction results in a temporal resolution approaching tens of femtoseconds^60^.

## Supplementary Information

Supplementary Information.

## Data Availability

The datasets generated and analysed during the current study are available in the Zenodo repository, 10.5281/zenodo.6365342.
