# Supplementary material for: Determination of sub-ps lattice dynamics in FeRh thin films
Source: Sci Rep. 2022 May 20;12:8584. doi: 10.1038/s41598-022-12602-w (PMC9122986; doi:10.1038/s41598-022-12602-w)
Supplement: Supplementary file 1 — Supplementary Information. [file 41598_2022_12602_MOESM1_ESM.pdf]

Determination of sub-ps lattice dynamics in FeRh thin films

Supplementary Information

Michael Grimes[1,2,3]‡, Hiroki Ueda[2], Dmitry Ozerov[2], Federico Pressacco[4,5], Sergii Parchenko[2,3], Andreas Asperos[2,3], Markus Scholz[4], Yuya Kubota[6], Tadashi Togashi[6,7], Yoshikazu Tanaka[6], Laura Heyderman[2,3], Thomas Thomson[1], and Valerio Scagnoli[2,3]†.

[1]NEST, Department of Computer Science, The University of Manchester, Oxford Road, Manchester, M13 9PL, UK.

[2]Paul Scherrer Institute, 5232 Villigen PSI, Switzerland.

[3]Laboratory for Mesoscopic Systems, Department of Materials, ETH Zurich, 8093 Zurich, Switzerland.

[4]Deutsches Elektronensynchrotron, DESY, Notkestrasse 85, D-22607 Hamburg, Germany.

[5]The Hamburg Centre for Ultrafast Imaging, Universität Hamburg, Mittelweg 177, 20148 Hamburg, Germany.

[6]RIKEN SPring-8 Centre, Sayo, Hyogo 679-5148, Japan

[7]Japan Synchrotron Radiation Research Institute (JASRI), 1-1-1 Kouto, Sayo, Hyogo 679-5198, Japan.

†valerio.scagnoli@psi.ch

‡michael.grimes@manchester.ac.uk

1. Experimental parameters

The pump-probe x-ray scattering experiment was conducted at BL3 of SACLA, Japan research facility. The scattering geometry, shown in the main text (Fig. 2a), was optimized to achieve similar laser and x-ray probe depths [1]. A nitrogen cryoblower was used to provide a stable temperature, minimising drift, over the period of the experiment. This also allowed us to change the ambient temperature of the sample to determine the effective temperature change that the laser pulse induces for a given fluence. The beamline was operated without monochromator [2] ($\Delta E/E \sim 6e^{-3}$), delivering photons with energies close to 6.408 keV. The x-ray energy was chosen to be below the Fe K-edge in order to minimize the background signal resulting from the excitation of Fe fluorescence. The instrument parameters for the x-ray diffraction measurements and laser excitation of the sample were:

(a) x-FEL pulse

Beam energy: E = 6.408 keV, ΔE = 0.040 keV (no monochromator, pink beam)

Beam size (⊥): Horizontal = 14.2 μm (FWHM), Vertical = 13.2 μm (FWHM) observed.

$\delta_P = (\lambda/2\pi\beta)*\sin\alpha \Rightarrow$ 100 nm @ $\alpha = 0.7°$, 50 nm @ $\alpha = 0.4°$

(b) Optical laser pulse

Beam spectrum: λ = 796.4 nm, Δλ = 37.4 nm

Beam size (⊥): Horizontal = 499.5 μm (FWHM), Vertical = 482.1 μm (FWHM)

Injection angle: 15.4° $\Rightarrow \delta_P \approx$ 31 nm

2. Estimation of x-ray diffraction parameters as function of pump-probe delay

In order to determine how the (-1 0 1) x-ray diffraction peak position and its FWHM evolve with time, we simulated the expected AF and FM peak intensity as a function of scattering angle. Based on the magnetometry results from Fig. 1c, we determined the ratio of each phase at a given temperature. The 1st derivative dM/dT can be fitted with a Gaussian function having a centre equal to T_T and a width equal to ΔT. We use this model based on previous experiments in the literature that have shown the transition is first order with a distribution of transition temperatures. The differences in transition temperatures can be seen on a microscopic scale which averages out to a Gaussian distribution across thin film samples [3].

For the thermal distribution model, we then assume the intensity of the AF and FM peaks are in direct proportion to the ratio of each phase. The peaks were modelled as Voigt functions with FWHM taken from the XRD data. The centre of each (-101) peak was found from the respective lattice constants (a_{AF} = 0.299 nm, a_{FM} = 0.302 nm [4]) subject to the diffraction condition, $n\lambda = d \cdot sin\theta$. We used the x-ray energy of the pink beam (ΔE = 40 eV ≈ 0.2°) available at SACLA (6.408 keV) for this calculation. From the sum of the two peaks, we extract the centre of mass (COM) and FWHM by matching the

experimental data to the model. Using an asymptotic heating profile to the final temperature, we were then able to replicate the time traces seen in the lattice dynamics in Fig. S1. This simulation confirms the assumption that the transition we observed was first order and the change in Bragg peak can be attributed to phonon heating of FeRh.

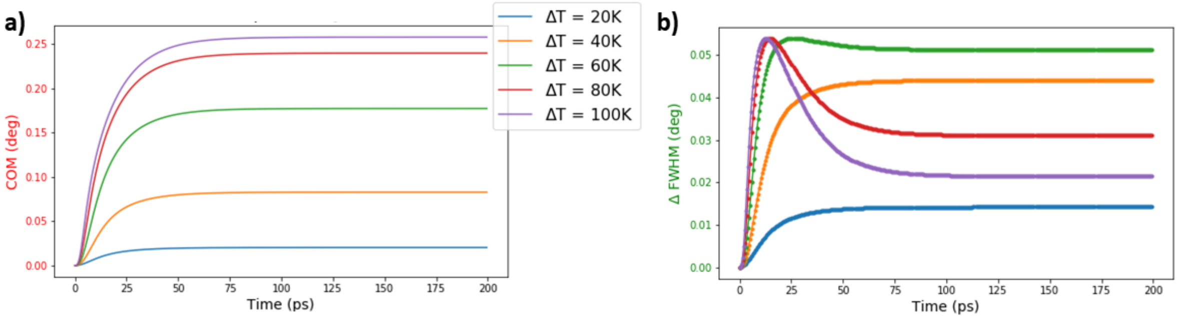

Figure S1 – Demonstration of the change of the centre of mass peak position (COM) and the change in the FWHM expected for the FeRh (-101) peak when the sample is excited by a given ΔT, assuming 1st order dynamics. This shows the increasing FHWM and peak shift as more of the sample is excited to the FM state. The characteristic shape of the ΔFWHM curve observed in the experimental data (see Fig. 5) emerges when sufficiently heated.

3. Extracting Debye-Waller factor from the static XRD measurements

In order to quantify the lattice expansion based on quasi-static XRD data, we required accurate estimates of the 2θ centre and peak FWHM. As the peaks do not show simple Voigt or Lorentzian shapes, a new model was devised. Following the work of Barton et. Al. [5], an inhomogeneous strain across the film was proposed to explain the asymmetric peak observed. In this work a shift in the 2θ angle of FeRh (002) is observed as the strain is relived. The data has a tail that appears to have a Voigt shape, with the leading edge composed of a series of strained contributions. This corresponds with a portion of the film being strained at the MgO interface [5]. Then a large portion of the film is unstrained yielding the tail of the peak, it should be noted that quasi-static XRD measurements probe the entire film thickness. To demonstrate the effect of inhomogeneous strain on the peak shape, Fig. S2 illustrates the expected peak shape for a film with a range of lattice constants, with increased contribution for the unstrained portions. This model could be adapted by changing n, the number of peaks with independent lattice constants. As n increases it approaches the behaviour of the continuum of strain as expected for the films presented in this work. The peaks in the Fig. S2a&b show the examples of the peaks for n=2 (solely AF and FM peaks), and the summation of the peaks for n=10 (strain profile).

In order to consistently treat the data, we only fitted to the tail of the data using a region with intensities 80% of the maximum and above as seen in Fig. S2c. This choice of cut-off was arbitrary but is seen to describe the tail end of the peak and therefore the unstrained film. This portion of the film is the main contributor to the peaks observed in the synchrotron experiment due to the grazing incidence geometry. Subject to this cut-off in 2θ, the static XRD peaks were fitted with Voigt functions in order to extract the position, width, and intensity. This allowed the lattice constant of the FeRh at each temperature to be determined.

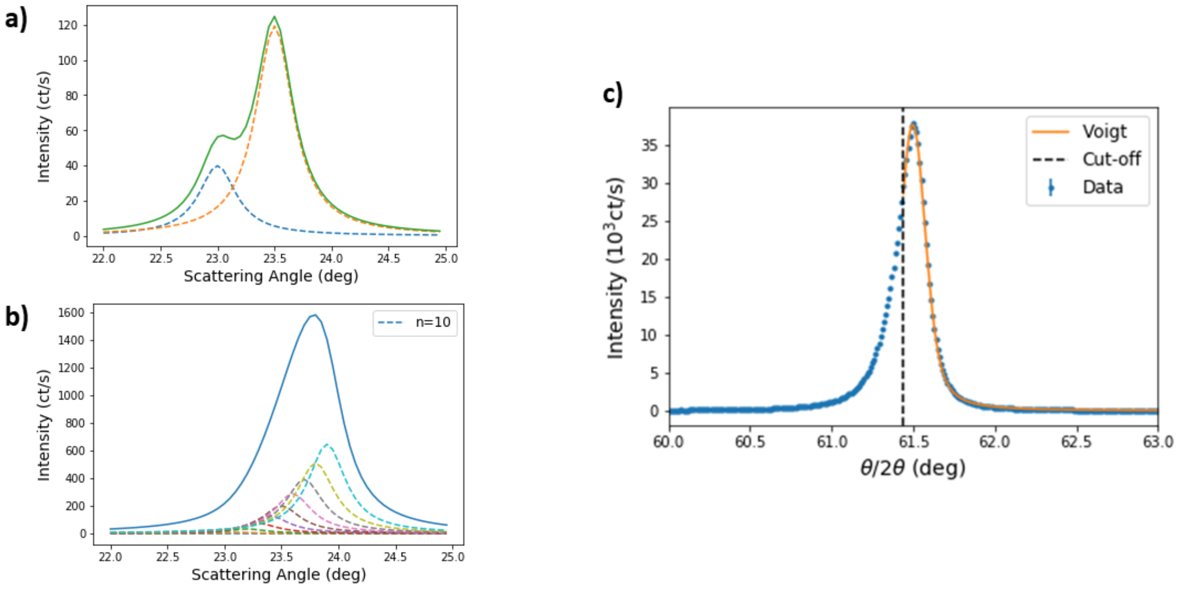

Figure S2 - Effect of strain on the XRD peak shape. a) Expected peak shape for two arbitrary overlapping peaks (dotted lines). The contribution of each peak can be seen in the sum (full line). b) We assume the strain profile produces a series of overlapping peaks to form the 'tailed' peak. The final 'strongest' peak indicates the unstrained peak and should therefore occur at the "bulk" 2θ position. c) XRD data fitted with a Voigt function when a cut off is applied according to the strain model discussed. The error bars, assuming Poisson statistics, are not visible on this scale.

By comparing the expansion to the RT value, the relative expansion was found. The substrate behaviour was found by fitting the MgO (002) peaks and used as a reference for the induced expansion. In order to plot Fig. 3b presented in the main paper, the following relation was used

$$\delta V\ (T) = \left[\frac{V_{FeRh}(T) - V_{FeRh}(RT)}{V_{FeRh}(RT)} \Big/ \frac{V_{MgO}(T) - V_{MgO}(RT)}{V_{MgO}(RT)} \right] \qquad (S.1)$$

Where $\delta V(T)$ is the change in volume (proportional to lattice constant), and $V_i(T)$ is the extracted volume at a given temperature from the (002) peak for the respective species. Using the values of integrated intensity extracted from the Voigt fit, we can estimate the Debye temperature of the

material and infer the lattice temperatures as a function of time [6] shown in the main paper, Fig. 7a. The Debye-Waller factor (DWF) presented in Eq. (1) & (2) of the main paper can be rearranged to provide the following estimate of the lattice temperature as a function of the scattering intensity

$$T_L = T_0 - \frac{M k_B \Theta_D^2}{3\hbar^2 q^2} \ln\left(\frac{I(T)}{I_0}\right) \qquad (S.2)$$

Where $I(T)$ is the peak intensity, and the remaining variables are defined as in Eq. (2) of the main text. We apply the treatment to the heated XRD peaks to find the pre-factor (χ_D) in the above equation. Fig. S3 shows the intensity of the static peaks as a function of temperature, using 423 K as T_0 as this was the most intense measured peak in Fig. 3a of the main text. The fitted line provides an estimate of the DWF for the FeRh XRD peaks.

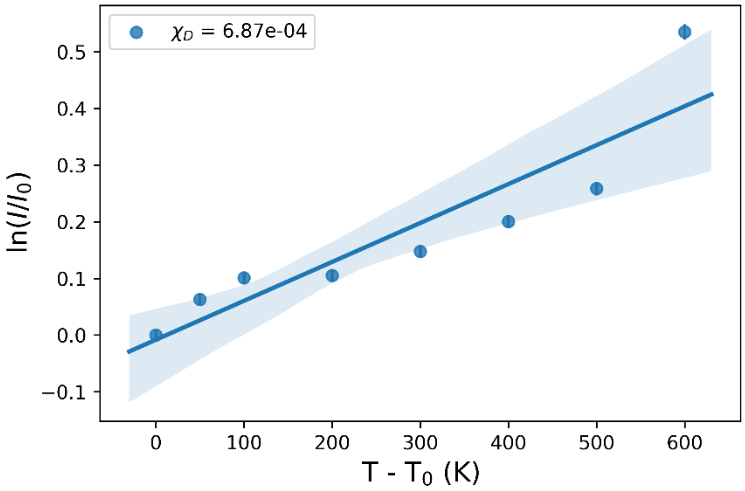

Figure S3 - Extraction of the DWF using the (002) peak intensity vs. temperature taken from the Rigaku XRD data of Fig. 3 in the main text, where T_0 is 473 K. This assumes the intensity is a direct probe of the relative amount of coherent scattering of the x-rays from the atomic centres. The 95% confidence bands are shown by the shaded region. Error bars refer to the uncertainty in the Voigt fitting of the integrated intensity.

Using this value of the DWF, we could plot the lattice temperature as a function of time as in the main text, Fig. 7. The pre-factor is changed to account for differences in the estimated average displacement for the (-101) peak. The same treatment is plotted for both different laser fluences and initial temperatures. This analysis provides a Θ_D = 234 ± 70 K, which is lower than that found in specific heat measurements. This is due to the Debye model of x-ray intensity only considering acoustic phonons in monatomic systems [7], so called Θ_M. By accounting for the crystal structure and species of FeRh, we predict an adjusted Θ_D = 331 ± 80 K, which agrees with *ab initio* predictions for the FM phase of 300 K [8].

4. Extension of the three-temperature model – transient phonon channel

The fitting the short (ps) time dependent diffraction data (Fig. 6) suggests a short-lived state in the phonon bands. By adding a term to the three-temperature model in the form of a new short-lived excited state the kinetics of the system can be explained. In the case of the higher laser excitation scans i.e., those above the 5.5 mJ cm^{-2} threshold fluence, this pathway becomes a significant part of the transition behaviour. To describe this behaviour, we first built a model based on solving differential equations from the three-temperature assumptions. In this model, the spin coupling is presumed to be relatively weak over the measured timescales. Due to the relatively long growth lifetimes of the magnetisation [9,10], the kinetics of the electron and phonon systems can be approximated as

$$\frac{d[T_{el}]}{dt} = -\frac{\gamma(T_{el} - T_l)}{C_{el}(T)}, \qquad (S.3)$$

$$\frac{d[T_l]}{dt} = \frac{\gamma(T_{el} - T_l)}{C_l(T)}. \qquad (S.4)$$

Where the subscript 'el' and 'l' refer to the electronic and lattice systems, respectively. *C* refers to the respective heat capacity and γ is the coupling strength. The change in system temperature as a function of time was numerically solved using an ordinary differential equation solver. The value for γ is estimated as the inverse of the extracted growth lifetime τ_G from Eq. (3) in the main text, and the heat capacity used was 3.20x10^{17} J m^{-3} s^{-1} K^{-1}. The heating of the sample from the laser is assumed to be due to the absorbed photons (non-reflected). The skin depth (δ_p), and the reflectance of the laser light are required to simulate the heating of the electronic system. These are found from the refractive index of FeRh at 800 nm [11], giving a value of δ_p=31 nm; while R=0.7 is found from reflectivity data [12]. In order to model the new highly excited system we adapted the equations to include the excited state by allowing the electronic system to relax via an intermediate channel [6].

$$2C_{el}(T)\frac{d[T_{el}]}{dt} = \frac{2(1-R)}{\delta_P}I(t) - \gamma^*(T_{el} - T_{l*}) \qquad (S.5)$$

$$\alpha C_{l*}(T)\frac{d[T_{l*}]}{dt} = \gamma^*(T_{el} - T_{l*}) - \gamma^1(T_{l*} - T_l) \qquad (S.6)$$

$$(1-\alpha)C_l(T)\frac{d[T_l]}{dt} = \gamma^1(T_{l*} - T_l) \qquad (S.7)$$

Where '*' refers to the transient lattice state. The laser heating parameters are captured in the first term of the electronic differential equation; $I(t)$ being the laser pulse intensity, R is the reflectivity, and δ_P is the skin depth and α is to the relative 'hot phonon' population when compared to the entire phonon system. The coupling between the electron and hot phonons is captured using γ^*, while coupling between excited and equilibrium states are implemented with γ^1. These can be estimated from the lifetimes τ_G and τ_G* extracted from the fitting performed in the main text, Fig. 6a.

Using previously published data on the calorimetric measurements on FeRh, we can plot the heat capacity of the electron and phonon systems of FeRh [13,14]. The latent heat of the transition is captured as a Gaussian function about the midpoint of the transition (355 K), with a value of 2.2 kJ kg^{-1} [13,15]. The non-equilibrium coupling between the electron and phonon systems is available for metals including Fe, but has been shown to vary by less than 5% over the temperature range [16], so a constant value was used. The parameters used in the model are presented in Fig. S4.

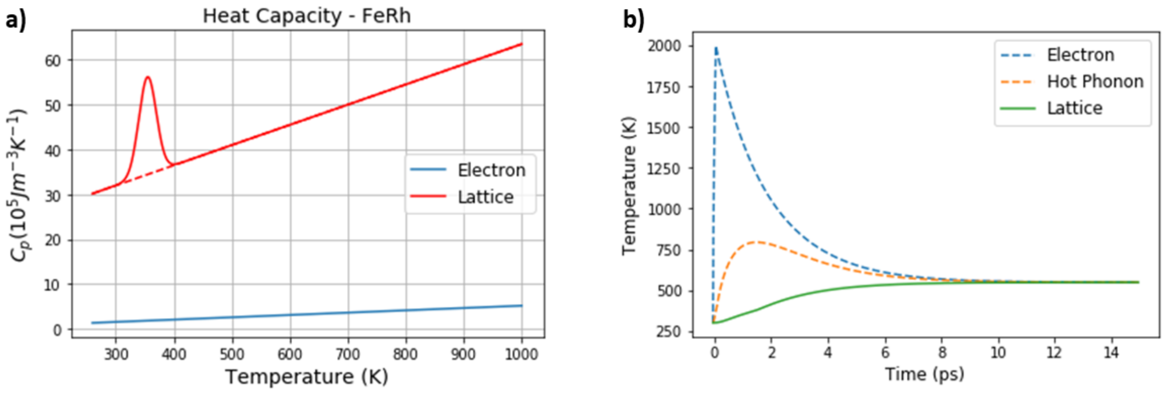

Figure S4 – a) Heat capacity of the FeRh electronic and lattice systems found using thermodynamic data from the literature [13–15]*. The latent heat of the transition is captured as a Gaussian peak in* C_L *about* T_T*. The electronic heat capacity is linear across this temperature range. b) The temperature of each subsystem as a function of time is presented following 7 mJ* cm^{-2} *laser excitation. The equilibrated state is reached within 10 ps.*

The effect of different fluences, shown in Fig. 7b of the main text, was simulated by increasing the intensity of the electron heating in Eq. (S.5). The difference between the higher energy system and the 'usual' lattice system is assumed to contract the lattice (PM phase) and the lattice temperatures is assumed to expand the lattice (AF-FM transition). We can see how the hot phonon phase can access the PM phonon bands on short timescales using this treatment of optical and acoustic phonons.

This model can be adapted to consider the ambient temperature. The effect of this is demonstrated in Fig. S5. It can be seen that the dynamics are slower at higher temperatures and fluences, as we have previously observed when fitting the transient intensity of the peaks. This can be understood by considering that the sample is driven further out of equilibrium when more energy is present in the system either in the form of thermal or laser excitation. This predicts that the peak shift is diminished for the lower ambient temperatures due to the reduced portion of film that enters the FM phase.

Overall, this model slightly overestimates the lattice heating at the lowest fluences and underestimates the high fluence lattice heating; with estimates of the maximum temperature reached

being 425, 537, 602 and 683 K for excitations of (2.9, 5.5, 7.1, 9.4 mJ cm^{-2}) for the simulations shown in Fig. 7b of the main text. We assume there are other forms of losses not accounted for in the model as we estimated that the lattice heating as a function of laser fluence is 40 K mJ^{-1} cm^2, slightly higher than previously reported values in the literature using similar pump laser regimes [9]. There may be other losses not considered as the model assumes any non-reflected light is absorbed by the system. With regard to the lowest fluence case, the model appears to be underestimating the latent heat for the AF → FM transition. This would explain why the model more closely predicts the behaviour when the laser fluences heat the material above the T_T (> 5 mJ cm^{-2}). The model could be improved by increasing the heat capacity around T_T which could be verified by calorimetric measurements on samples with a range of T_T values which has not been explored experimentally to date. The high fluence cases can be explained by considering the intensity, which was assumed to be a direct probe of the lattice temperature. At the highest fluences, the intensity is further decreased by the shift in 2θ caused by the lattice expansion meaning the extracted lattice temperature is slightly increased.

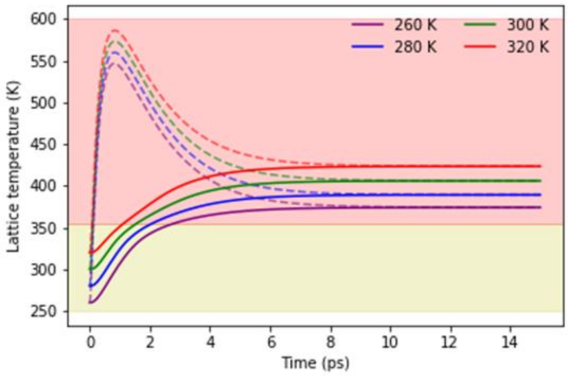

Figure S5 - Estimation of the lattice temperature evolution both for the bulk phonon bands (full lines) and the highly coupled hot-phonons (dotted lines) as a function of pump-probe delay. The effect of different ambient temperatures is shown for the lowest fluence case used in the experiment (2.9 mJ cm^{-2}) . Equilibration of the phonon temperatures is achieved within 10 ps in all cases.

In general, this model predicts slightly faster than observed changes of peak intensity and higher equilibrium temperatures for both reduced fluences and lower ambient temperatures. However, this is a good first approximation for the non-trivial electron-phonon coupling as the approach used is mainly based on theoretical studies of the FeRh.

References

1. Grimes, M. *et al.* X-ray investigation of long-range antiferromagnetic order in FeRh. [In press]. *AIP Adv.* (2022).

2. Tono, K. *et al.* Beamline, experimental stations and photon beam diagnostics for the hard x-

ray free electron laser of SACLA. *New J. Phys.* **15**, 083035 (2013).

3. Warren, J. L., Barton, C. W., Bull, C. & Thomson, T. Topography dependence of the metamagnetic phase transition in FeRh thin films. *Sci. Rep.* **10**, 4030 (2020).

4. Zsoldos, L. Lattice Parameter Change of FeRh Alloys due to Antiferromagnetic-Ferromagnetic Transformation. *Phys. status solidi* **20**, 25–28 (1967).

5. Barton, C. W. *et al.* Substrate Induced Strain Field in FeRh Epilayers Grown on Single Crystal MgO (001) Substrates. *Sci. Rep.* **7**, 44397 (2017).

6. Mansart, B. *et al.* Temperature-dependent electron-phonon coupling in La2-xSr xCuO4 probed by femtosecond x-ray diffraction. *Phys. Rev. B - Condens. Matter Mater. Phys.* **88**, 054507 (2013).

7. Horning, R. D. & Staudenmann, J. The Debye–Waller factor for polyatomic solids. Relationships between X-ray and specific-heat Debye temperatures. The Debye–Einstein model. *Acta Crystallogr. Sect. A* **44**, 136–142 (1988).

8. Jiménez, M. J., Schvval, A. B. & Cabeza, G. F. Ab initio study of FeRh alloy properties. *Comput. Mater. Sci.* **172**, 109385 (2020).

9. Mariager, S. O. *et al.* Structural and magnetic dynamics of a laser induced phase transition in FeRh. *Phys. Rev. Lett.* **108**, 087201 (2012).

10. Pressacco, F. *et al.* Laser induced phase transition in epitaxial FeRh layers studied by pump-probe valence band photoemission. *Struct. Dyn.* **5**, 034501 (2018).

11. Chen, L.-Y. Optical properties of metals and alloys: Au, Ag, FeRh, AuA12, and PtA12. (Digital Repository @ Iowa State University, http://lib.dr.iastate.edu/, 1987). doi:10.31274/rtd-180813-12939

12. Bennett, S. P., Currie, M., van 't Erve, O. M. J. & Mazin, I. I. Spectral reflectivity crossover at the metamagnetic transition in FeRh thin films. *Opt. Mater. Express* **9**, 2870 (2019).

13. Cooke, D. W. *et al.* Thermodynamic measurements of Fe-Rh alloys. *Phys. Rev. Lett.* **109**, 255901 (2012).

14. Richardson, M. J., Melville, D. & Ricodeau, J. A. Specific heat measurements on an Fe Rh alloy. *Phys. Lett. A* **46**, 153–154 (1973).

15. Nikitin, S. A. *et al.* Giant elastocaloric effect in FeRh alloy. *Phys. Lett. A* **171**, 234–236 (1992).

16. Lin, Z., Zhigilei, L. V & Celli, V. Electron-phonon coupling and electron heat capacity of metals under conditions of strong electron-phonon nonequilibrium. *Phys. Rev. B - Condens. Matter Mater. Phys.* **77**, 075133 (2008).
